# Experimental Infection of Sheep at 45 and 60 Days of Gestation with Schmallenberg Virus Readily Led to Placental Colonization without Causing Congenital Malformations

**DOI:** 10.1371/journal.pone.0139375

**Published:** 2015-09-29

**Authors:** Ludovic Martinelle, Antoine Poskin, Fabiana Dal Pozzo, Nick De Regge, Brigitte Cay, Claude Saegerman

**Affiliations:** 1 Research Unit of Epidemiology and Risk Analysis Applied to Veterinary Sciences (UREAR-ULg), Fundamental and Applied Research for Animals & Health (FARAH), Faculty of Veterinary Medicine, University of Liège, Liège, Belgium; 2 Operational Directorate Viral Diseases, Veterinary and Agrochemical Research Centre (CODA-CERVA), Brussels, Belgium; The Pirbright Institute, UNITED KINGDOM

## Abstract

**Background:**

Main impact of Schmallenberg virus (SBV) on livestock consists in reproductive disorders, with teratogenic effects, abortions and stillbirths. SBV pathogenesis and viral placental crossing remain currently poorly understood. Therefore, we implemented an experimental infection of ewes, inoculated with SBV at 45 or 60 days of gestation (dg).

**Methodology:**

“Mourerous” breed ewes were randomly separated in three groups: eight and nine ewes were subcutaneously inoculated with 1 ml of SBV infectious serum at 45 and 60 dg, respectively (G45 and G60). Six other ewes were inoculated subcutaneously with sterile phosphate buffer saline as control group. All SBV inoculated ewes showed RNAemia consistent with previously published studies, they seroconverted and no clinical sign was reported. Lambs were born at term via caesarian-section, and right after birth they were blood sampled and clinically examined. Then both lambs and ewes were euthanatized and necropsied.

**Principal Findings/Significance:**

No lambs showed any malformation suggestive of SBV infection and none of them had RNAemia or anti-SBV antibodies prior to colostrum uptake. Positive SBV RNA detection in organs was rare in both G45 and G60 lambs (2/11 and 1/10, respectively). Nevertheless most of the lambs in G45 (9/11) and G60 (9/10) had at least one extraembryonic structure SBV positive by RTqPCR. The number of positive extraembryonic structures was significantly higher in G60 lambs. Time of inoculation (45 or 60 dg) had no impact on the placental colonization success rate but affected the frequency of detecting the virus in the offspring extraembryonic structures by the time of lambing. SBV readily colonized the placenta when ewes were infected at 45 or 60 dg but infection of the fetuses was limited and did not lead to congenital malformations.

## Introduction

In summer 2011, a new unspecific clinical syndrome was first described in adult cattle in Germany causing febrile disease, milk drop and diarrhoea [1], later attributed to a novel *Orthobunyavirus* (family *Bunyaviridae*) named Schmallenberg virus (SBV). Palearctic telmophagous midges of the genus *Culicoides* [2–4] were identified as the vector of SBV. However the most striking consequence of SBV spreading throughout Europe was the epizootic of malformations in ruminant offspring, leading to abortions, peripartum mortality and stillbirths. Indeed, SBV was linked to an arthrogryposis / hydranencephaly syndrome in ruminant newborns following *in utero* infection [5].

According to literature about Akabane virus (AKAV), a closely related *Orthobunyavirus*, the moment of the gestation when the mother gets infected influences the outcome of an *in utero* infection. In sheep, Parsonson et al. suggested that AKAV might only be able to reach the fetus if placenta is developed and vascularized enough [6], whereas Hashiguchi et al. considered congenital malformations highly unlikely to occur if infection took place after 50 days post insemination [7]. So far, susceptible gestation periods leading to congenital malformations need to be clarified in the different SBV host species.

In several European countries, SBV had a greater impact on sheep flock than in cattle herds [8, 9]. As a matter of fact, dairy cattle give birth basically all year round while lambing are mostly seasonal, with mating of the sheep concentrated in July-August or October-November, depending on the breed [10]. Indeed, sheep breeding seasons are widely overlapping with some of the highest vector activity periods in Europe [11]. Moreover, increased odds of malformations were reported in sheep flock with an early mating season in 2011 [12]. Thus considering the breeding season as a risk factor in correlation with the vector activity, the management of the breeding season could be a key element to avoid congenital malformations and to get ewes infected before or after the critical period of susceptibility for the fetus. Based on the pathogenesis of Akabane virus and epidemiological studies of the SBV outbreaks, it is assumed that teratogenic infection takes place in the first trimester. Stockhofe et al. performed SBV experimental infection of sheep at 38 and 45 days of pregnancy [13], however the infected ewes were slaughtered one week after inoculation. The aim of the current study was to investigate the occurrence of malformation at term in ewes infected later in the course of gestation. Thus we implemented an experimental study performed on groups of pregnant ewes subcutaneously infected with infectious SBV serum at day 45 and 60 of gestation. Therefore we could get a better insight on SBV pathogenesis in pregnant ewes and rate of transplacental transmission and colonization following two different gestation times of inoculation.

## Materials and Methods

### Ethical statements

The experiments, maintenance and care of ewes complied with the guidelines of the European Convention for the Protection of Vertebrate Animals used for Experimental and other Scientific Purposes (CETS n° 123). The protocol used in this study was approved by the Ethical Committee of the IPH-VAR (Scientific Institute of Public Health—Veterinary and Agrochemical Research Center (VAR), number of project: 121017–01) on the 11^th^ February 2013. All surgery was performed using xylazin (Paxman®, Virbac, France) and local anesthesia (procaine hydrochloride 4%, VMD, Belgium), and all efforts were made to minimize suffering.

### Animals

A total of 23 “Mourerous” ewes of about 1 year-old and originating from a SBV free area in France were used in this experiment (original sheep flock from the *Alpes-Maritimes* department (ISO code FR-06), animals selected after a last serological screening carried out on the 08/11/2012). The Mourerous is a middle-size rustic breed from south of France. All the animals were serologically and virologically negative for SBV as determined by ELISA, SNT and RTqPCR (see below) before and after arrival at the experimental animal centre of CODA-CERVA where they were kept in Biosafety Level 3 facilities. The ewes also tested negative for bluetongue virus (BTV) and Maedi-Visna virus. The animals were only implemented in the experiments after a thorough clinical examination to ensure their asymptomatic state and good clinical condition in range with the physiological parameters [14].

### Inoculum

The infectious serum used for inoculation of the sheep was obtained from Friedrich Loeffler Institute (Riems, Germany) and was already successfully tested in calves and sheep [1, 15, 16]. Briefly, the infectious serum originated from a heifer sampled two and three days after inoculation with infectious whole blood from a SBV-positive cow. The inoculum contained about 2 x 10^3^ 50% tissue culture infective dose/ml (TCID_50_/ml) [15] and 7.3 x 10^6^ RNA copies/ml of SBV S-Segment as determined by a RTqPCR detecting the SBV-S segment (see below).

### Insemination and diagnosis of gestation

The animals were synchronised using Veramix sponges (60 mg Medroxyprogesterone Acetate, Zoetis®, Louvain-La-Neuve, Belgium). The sponges were removed 12 days after insertion and the ewes received 500 IU Pregnant Mare Serum Gonadotrophin through intramuscular route. Intracervical artificial insemination (AI) has been realized 52 hours after sponge removal, with a double dose of semen.

A total of four rams, from 1 to 6 years old, were used to get semen doses: 3 *Ile-de-France* and one crossbred Texel X Ile-de-France. These rams originated from a Maedi-Visna negative flock, and despite being SBV seropositive, their semen has been tested negative twice, one week apart, for SBV RNA (RTqPCR). A sample of each of the semen doses used for the actual AI has been also tested negative for SBV RNA *a posteriori*.

Gestation diagnostic has been performed by detection of pregnancy associated glycoprotein (PAG) by radio immune-assay (RIA) at 27 days post AI as previously described [17] and then by ultrasonography at 40/55 and 95/110 dg for the groups inoculated at 45 and 60 dg, respectively.

### Experimental design

During the whole duration of the experiment insect light/glue traps were displayed and regularly removed and replaced, allowing identification of the caught insects to ensure the absence of *Culicoides*.

The ewes were inoculated subcutaneously (SC) in the left axilla with 1 ml of infectious serum. Eight ewes were inoculated at 45 dg (G45, ewe1 to ewe8) and nine ewes at 60 dg (G60, ewe9 to ewe17). Two control groups of three animals each were inoculated SC with sterile phosphate buffer saline respectively at 45 and 60 dg (control G45 and control G60, ewes18 to 20 and ewes21 to 23).

During the first two weeks of the experiment, clinical, virological and serological monitoring was realized on a daily basis, then once a week until the end of the experiment.

At the expected lambing period for each respective group, ewes were closely monitored and when first signs of labor could be identified, the ewe was anesthetized with xylazin (Paxman®, Virbac, France) intravenously (IV) and local anesthesia (procaine hydrochloride 4%, VMD, Belgium), and lambing was perform by Caesarian section. Once the Caesarian section was realized, the ewe was euthanatized with IV injection of 5 ml of T61® (MSD Animal Health BVBA, Belgium) and subsequently necropsied.

Lambs were identified following their mother (ewe1 gives birth to lamb1; plus a, b or c in case of multiple gestation). When the lambs were born alive they were revived to allow the evaluation of any neurological trouble. Ability of standing up and suckling reflex were evaluated and a morphologic examination, with special emphasis on skull and limbs, was also performed. Blood samples were taken and lambs were then euthanatized with 1 ml T61® IV and necropsied. Tissue samples were taken from extraembryonic structures (intercotyledonary membrane, placentome, umbilical cord, amniotic fluid, meconium), and organs including CNS (brain, cerebellum, brainstem, spinal cord), lymphoid organs (prescapular, mesenteric, submandibular, mediastinic lymphnodes, thymus and spleen), other inner organs and tissues (gonads, adrenal gland, liver and lung) and musculoskeletal structures (femoral cartilage, *Musculus Semitendinosus*) for viral RNA detection. Placentomes were collected, longitudinally cut and separated in two halves for subsequent SBV RNA detection. The Intercotyledonary membrane was sampled in the area surrounding the collected placentomes. Samples (2 cm long) of the umbilical cord were collected 4–5 cm distal to the newborns.

### SBV detection by RTqPCR

The extraction of RNA from organs, feces, blood and serum and the detection of SBV-S segment by a one-step RTqPCR were performed as previously described [18].

The Cq values were converted into S-segment copy numbers using a RNA standard curve that was tested in each PCR run [19]. Briefly, RNA was extracted from SBV infectious serum with the RNeasyMini kit (Qiagen) following manufaturer’s instructions and reverse transcribed with M-MLV Reverse Transcriptase (Life Technologies). A PCR amplification using primers targeting a 839bp fragment of the S gene of SBV (forward: 5’-CTAGCACGTTGGATTGCTGA-3’; reverse: 5’-TGTCCTTGAGGACCCTATGC-3’; Integrated DNA Technologies) was performed using the FastStart PCR Master kit (Roche). The fragment was cloned in the 2.1-TOPO cloning vector (Life Technologies) and transformed into competent *Escherichia Coli* TOP10 cells and multiplied. The plasmids were isolated and then linearized with BamHI, followed by *in vitro* transcription with the TranscriptAid T7 High Yield kit (Thermo Scientific) following manufacturer’s instructions. Remaining DNA plasmids were eliminated by 2 successive Turbo DNA free treatments (Life Technologies) following manufacturer’s instructions and the RNA was purified using the RNeasyMini kit (Qiagen). The copy number was calculated based on the predicted molecular weight of the RNA transcripts. Aliquots of the RNA transcripts were stored at −80°.

The RNA standard curve consisted of a ten-fold serial dilution of the RNA transcripts in TE buffer. The dilution series ranging from 3.9×10^7^ to 3.9 copies/μL were run together with samples of blood or organ and the standard curve was constructed by plotting the Cq values against the log of the input RNA copy number. A linear regression was fitted to the scattered points and was used to calculate the number of copies in the samples ran during the same RTqPCR.

### Serology

The presence of neutralizing anti-SBV antibodies was assessed by seroneutralization (SNT), following the method described by De Regge et al. [20] and using an SBV isolate obtained from brain tissue of a lamb aborted in Belgium in 2011 which was passaged four times in Vero cells. Two positive and one negative control were added systematically to each assay. The titer was determined as the reciprocal of the highest serum dilution in which the entire monolayer was still intact. Sera were considered positive if the titer was ≥4 (specificity of 100% [20]).

### Statistical analysis

Differences in RNAemia of the different groups through time were assessed by two-way ANOVA with repeated measures. Difference between mean maximum copy numbers by group was evaluated with Welch test for unequal variance. Differences in positive detection of viral RNA in lambs, organs and reproductive performances (viability at birth, prolificacy) were evaluated by Chi² test or Fisher’s exact test for count data depending on the sample size.

*P* values ≤ 0.05 were considered significant. Statistical analyses were performed using the R software/environment (R-3.0.1, R Foundation for Statistical Computing, http://www.r-project.org/).

## Results

### Clinical impact and lambing

No clinical impact following inoculation was measured in ewes. The temperatures stayed within the physiological range upon euthanasia. All the animals conserved a good appetite and remained in good general condition.

Table 1 reports sex ratio, prolificacy rate and the percentage of living lambs at birth. No significant different was reported in the viability of the offspring between infected groups and between infected and control groups (Chi² test = 0.7, *P* = 0.4 and Chi² test = 0.3, *P* = 0.6, respectively; df = 1). Prolificity rate was also not significantly different between infected groups and between infected and control groups (Chi² test = 0.1, *P* = 0.74 and Chi² test = 0.008, *P* = 0.93, respectively; df = 1). None of the lambs had malformation evocative of SBV infection (arthrogryposis-hydranencephaly syndrome, stiff neck, scoliosis, brachygnathism). Only one lamb was born three days before the expected date and was in good health. All other lambs were born at term, no malformation was observed and they were able to stand up and showed a good suction reflex.

**Table 1:** Reproductive performances.

Group	Number of lambs	Sex ratio	Weight at birth (Kg)^a^	Prolificacy rate (%)	% of healthy living lambs
G45	11	0.8	4^a^(0.7); 3.1^b^(1.1); 2^c^(0.3)	137.5	63.6
G60	10	1.5	4^a^(0.5); 3.3^b^(0.5)	111.1	80.0
Control G45	4	1.0	5^a^(1)	133.3	50.0
Control G60	3	2.0	4.5^a^(0.5); 3^b^(1.8)	100.0	67.0
**Total**	28	1.2	3.7(1.2)	121.7	67.9

Sex ratio is the ratio of male lambs to female lambs. Prolificacy is defined as the number of progenies born per parturition.

^a^: Single lambs

^b^: Twins

^c^: Triplets

Standard deviation follows in curved brackets.

### SBV RNA detection

#### SBV detection in the blood and organs of the ewes

**Fig 1:**
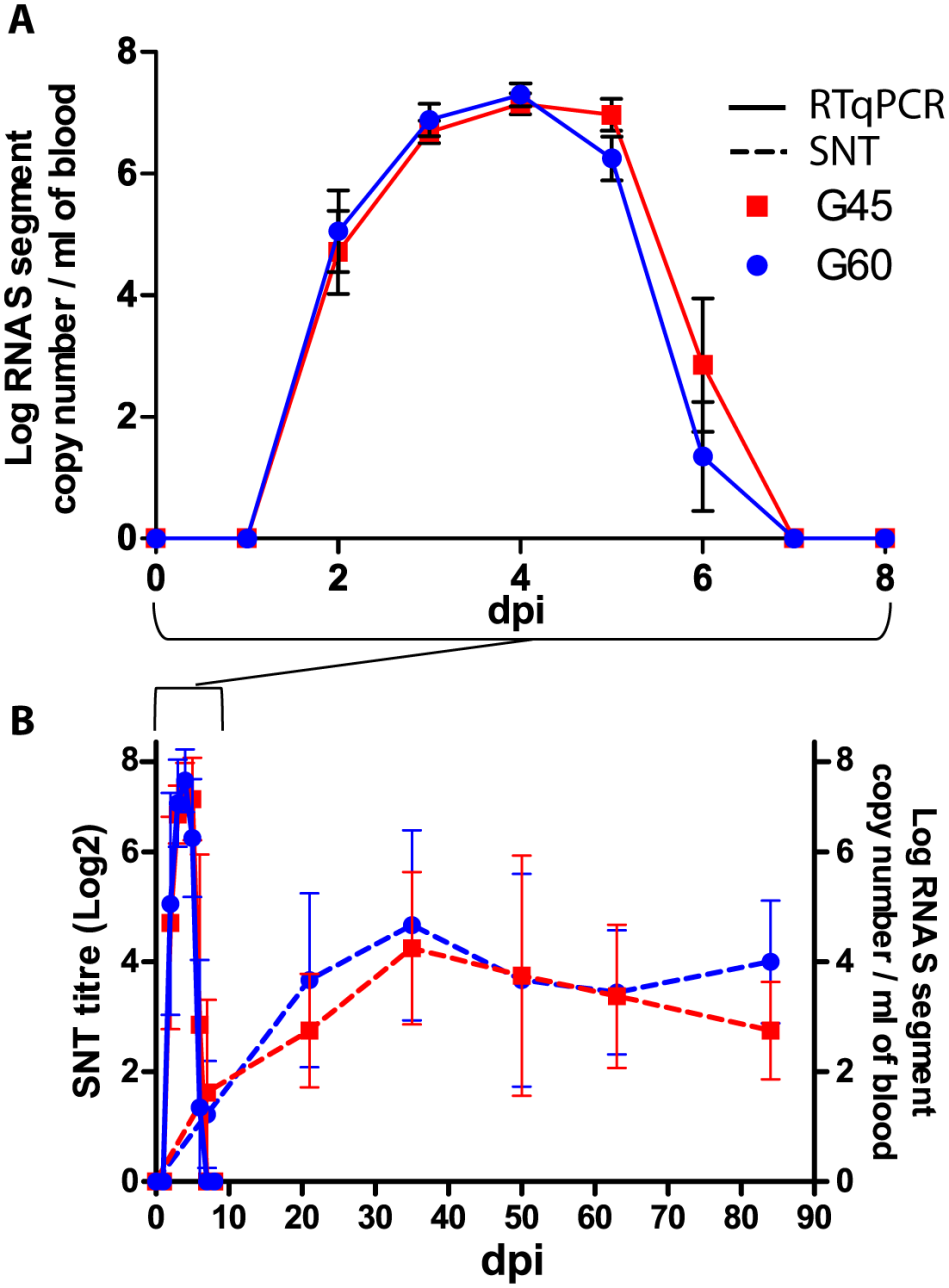
SBV viral RNA detection and neutralizing antibodies detection in the serum of the pregnant ewes. Detection of SBV genome in serum for G45 and G60 ewes (A). Results of SNT for G45 and G60 ewes and SBV genome detection downscaled to 90 days (B). Full line represents SBV genome detection and dashed line represents SNT.

No viral RNA was detected in the blood of the ewes from the control groups. SBV genome was first detectable at 2 dpi in both G45 and G60 groups and could be detected for maximum 5 days (Fig 1). The mean SBV genome copy numbers detected in the blood of sheep with RNAemia were not significantly different between G45 and G60 groups through time (two-way ANOVA for repeated measures; group effect: *P* = 0.4; group-time interaction: *P* = 0.44). The mean of the maximal SBV RNA copy number was not significantly different between G45 and G60 (Welch test for unequal variance; *P* = 0.83).

SBV genome could be detected in the ovaries of ewe9, in the ovaries and the spleen of ewe11 and only in the spleen ewe16, all belonging to G60. No SBV nucleic acid could be detected in any of the G45 ewes or control groups’ organ samples.

#### SBV detection in the blood and organs of the lambs

No SBV RNA could be detected in the blood of any of the lambs, from any of either the infected or control groups.

**Fig 2:**
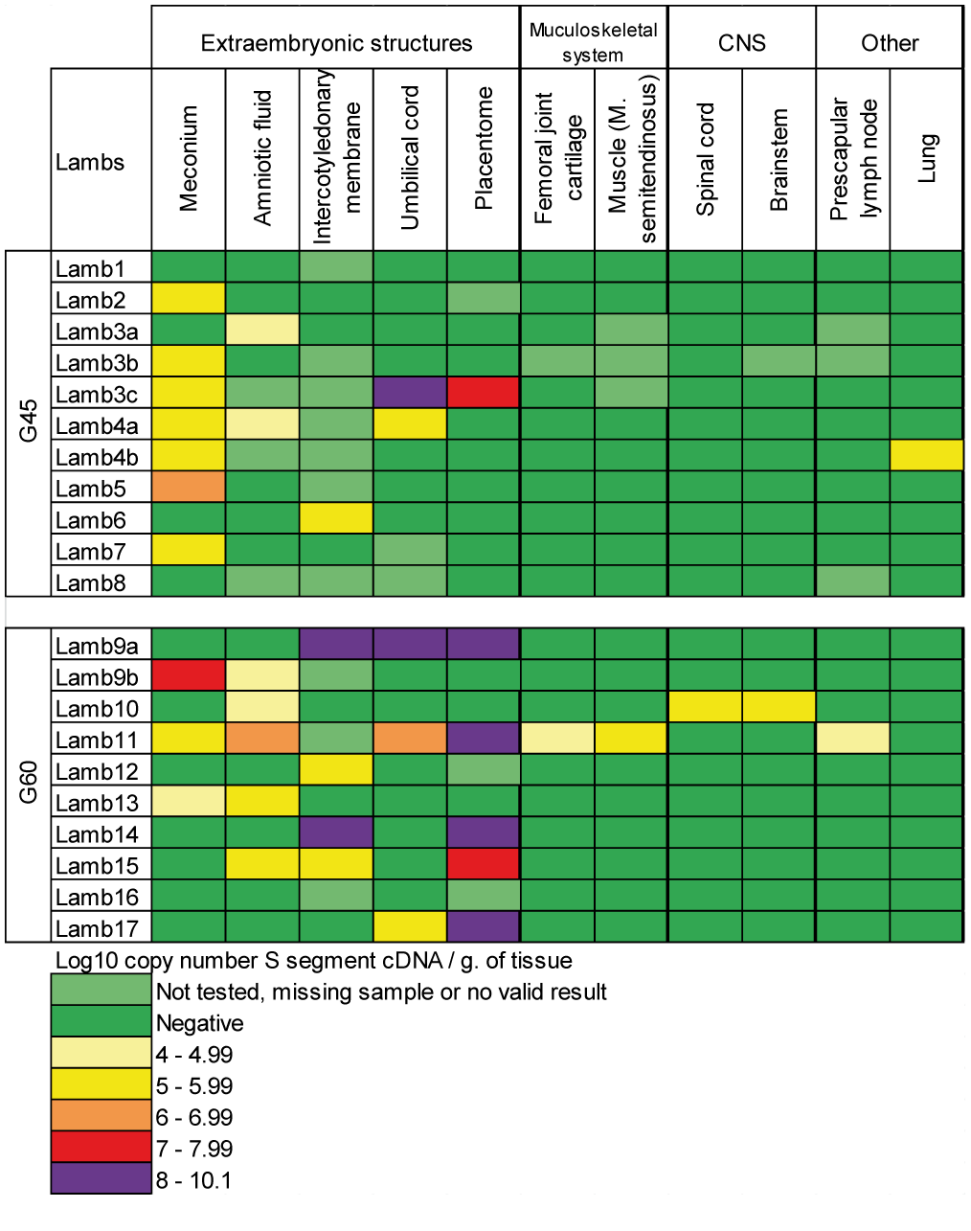
Mondrian matrix of SBV viral RNA detection in organs at necropsy. Rectangles color-coded according to SBV detection level. CNS: Central nervous system.

SBV RNA was shown in the brainstem and spinal cord of lamb10 and in the femoral joint cartilage, prescapular lymph node and muscle *M*. *semitendinosus* of lamb11 (both G60); in G45 viral genome could only be demonstrated in the lung of lamb4b (Fig 2).

#### SBV detection in extraembryonic structures

Intercotyledonary membrane was the most frequently SBV positive extraembryonic structure in both G45 and G60, (50% of tested samples; 5/10) followed by amniotic fluid (38,9%; 7/18), umbilical cord (38.5%; 5/18) and placentomes (33.3%; 6/18) (Fig 2). Percentage of positive detection was higher in G60 (47.2%; 17/36) versus G45 (19.4%; 6/31) for each of the latter structures, and overall difference was significant (Chi² test = 5.74, df = 1, *P* = 0.017). For individual extraembryonic structure, detection was only significantly more frequent in G60 (55.6%; 5/9) versus G45 (10%; 1/10) for placentome (Chi² test = 4.55, df = 1; *P* = 0.033). Meconium of G45 lambs was found more often positive than G60 ones (7/11 versus 3/10), but that difference was not significant (Chi² test = 3.38, df = 1, *P* = 0.12).

The rate of placenta colonization, as defined as the number of lambs with at least one positive extraembryonic structure divided by the total number of lamb, was however not significantly different between G60 (90%; 9/10) and G45 (82%; 9/11), respectively (Fisher’s exact test for count data = 1.94; *P* = 1).

### Serology

All the inoculated ewes seroconverted within three weeks, with first positive detection at 7 dpi for several ewes of both G45 and G60. Then all the inoculated ewes remained seropositive until the end of the experiment (Fig 1B).

No newborn lamb had anti-SBV neutralizing Abs before colostrum uptake.

## Discussion

Viral placental crossing is defined as the passage of the virus from the mother through the placenta to fetal tissues. Thus, placental colonization does not necessarily imply passage of the pathogen to the fetus itself [21]. In this study SBV placental colonization could be demonstrated after experimental infection in most of pregnant ewes at 45 and 60 days of gestation. Extraembryonic structures were found frequently positive, as previously described after natural infection [18, 20].

However no teratogenic effect could be reproduced. Congenital malformations caused by SBV remain a rare phenomenon in the field; teratogenesis associated with SBV infection has been observed only in 1 malformed calf out of 72 ones born from infected dams in natural conditions [22]. In addition, Veldhuis et al. (2014) estimated in cattle the malformation rate at about 0.5% of infected fetuses [23]. Therefore with respect to the number of newborn lambs considered in the current study, the lack of malformations is not especially surprising, despite lambs being thought to display more often malformations caused by SBV than calves [9, 24–25].

In both G45 and G60, the percentage of successful placental colonization was high (9/11–82% and 9/10–90%, respectively) and consistent with previous experimental data reporting a 100% placental colonization with or without placental crossing in ewes infected with SBV at 38 or 42 dg [13]. However, in our study significantly more positive samples, from both extraembryonic structures and lamb organs, were found in newborn lambs originating from ewes that were infected at 60 dg compared to those infected at 45 dg. It is likely that infection in most of G45 fetuses was about to be resolved with SBV eliminated from many organs whereas G60 fetuses could not clear the virus as much, the infection being more recent in the latter. Indeed the G60 group had a shorter period to eliminate the virus (85–87 days for G60 instead of 100–102 for G45). Another explanation could derive from the hypothesis emitted by Parsonson et al. regarding AKAV, a close related *Orthobunyaviruses*. These authors hypothesized the efficiency of the placental crossing being related to the development and vascularization of the placentomes, possibly explaining the highest detection rate in G60 when compared to G45 [6]. The higher development of placentomes at 60 dg might provide more target cells to infect and therefore lead to prolonged positive detection in the infected tissues. According to Parsonson et al., fetal trophoblastic cells constitute privileged target cells for AKAV, and SBV was reported to readily replicate in ovine trophoblast cells both *in vitro* [26] and *in vivo* [8], possibly being important target cells for SBV as well. Lawn et al. [27] reported that in sheep placenta examined at 42 days of gestation, placentomes are rather undifferentiated with only a few displaying rudimentary villi and crypts. Therefore placentomes at 45 days of gestation might be insufficiently developed to support intensive viral replication, yet allowing placental colonization. By contrast, placentomes at 60 days of gestation could better sustain SBV replication, as reported for AKAV [6].

A strong correlation between malformations and precolostral anti-AKAV antibodies has been reported [28], and growing evidences suggest that this pattern is shared by SBV, with 79 to 91% of naturally infected malformed lambs reported to be seropositive before colostrum uptake [29, 30]. Therefore in the current study, with no lamb showing SBV-associated abnormalities it is not surprising that all the precolostral sera were negative.

To produce Abs, the fetus has to be immunocompetent and the virus has to reach the systemic blood circulation of the fetus. In sheep *in utero* acquisition of humoral immune competence can be roughly estimated to span from 66 to about 100 dg [31]. For AKAV production of neutralizing antibodies is considered to start at 65–75 dg in sheep [32]. However Parsonson et al. reported ewes infected between 30 and 36 dg giving birth to malformed lambs with precolostral neutralizing antibodies against AKAV [33]. In the latter study a significant individual variation in humoral response of fetuses to AKAV was reported and no stage of gestation could be associated to seroconversion in 100% of the infected animals.

Therefore, despite a frequent successful placental colonization of SBV, infection of the fetus itself might be far less common, since SBV RNA could only be detected in some organs of lamb4b (G45), lamb10 (G60) and lamb11 (G60). Together the rare productive infection of the fetus and the individual variation could explain the absence of precolostral Abs against SBV in this study.

Although SBV RNA was detected at least once in almost all the extraembryonic structures of the lambs born from infected ewes, SBV positive detection in central nervous system (CNS) was very scarce with only one brainstem and one spinal cord sample found positive from lamb10 (G60 lamb). In ruminant newborns showing congenital defects suggestive of SBV infection, CNS is very often positive by RTqPCR [18, 20]. Here, despite SBV detected in several organs but CNS (with the exception of lamb10 with SBV RNA in spinal cord and brainstem), no malformations were reported, supporting a CNS invasion and SBV genome detected at birth possibly being associated to congenital malformations.

From the infected pregnant ewe to the CNS of the fetus the virus has to cross two major histological obstacles, namely placenta and blood-brain barriers (BBB).

It has been suggested that the development of the BBB was the reason that adult animals only show asymptomatic or very mild SBV infection [26]. In sheep BBB to sucrose (small and metabolically inert molecule) starts to develop around 50–60 dg, undergo a large decrease of permeability at about 70 dg and keep developing until 123 dg [34]. Nevertheless no malformed lambs were found in G45 and G60 although G45 ewes were infected before the assumed establishment of the BBB. Orthobunyaviruses are considerably larger (about 100 nm in diameter [35]) when compared to sucrose (0.44–0.53 nm); hence viral particles crossing might be hindered already at an earlier stage of gestation. A delayed transmission from the mother to the fetus is unlikely since Stockhofe et al. reported a 100% placental crossing 7 days after the infection of pregnant ewes with SBV [13]. The critical period of gestation leading to malformation in lambs might be narrower, possibly closer to the lower estimate for AKAV, from 28 to 36 dg [8], or to the one reported by Sedda et al. for SBV, between 37 and 42 dg [36]. Herder et al. [37] described SBV antigens in ruminants CNS mostly detected in temporal and parietal lobes, mesencephalon and hippocampus. Besides, cerebellar hypoplasia was frequently reported. Several critical neurologic events related to these structures occur during the first third of gestation in sheep: post-proliferative zone appears in the cerebellum between 21 and 31 dg; hippocampus CA1/CA3 neurogenesis spans from 22 dg to 59 dg, and neurogenesis of the cortical layer VI peaks around 40 dg [38]. Moreover in the spinal cord motor neurons are differentiated and develop mostly between 31–35 dg, corresponding to the time the embryos become motile [39]. Thus most of these decisive steps were over or about to be finished by 45 dg. As a consequence SBV infecting fetuses after 45 dg might be able to cross the BBB but might fail to invade CNS because of the scarcity of developing target cells.

It was assumed that SBV induced malformations are secondary to CNS lesions since SBV shows a clear tropism for nervous tissues [29, 33]. Infection by pestiviruses like border disease virus and bovine viral diarrhea virus are characterized by CNS dysplasia and hypomyelinogenesis in a similar way than following AKAV infection [40]. However since arthrogryposis induced by pestiviruses is neither as frequent nor as severe than in cases caused by AKAV infection, authors hypothesized that the increased frequency of musculoskeletal troubles following *in utero* AKAV infection might be related not only to CNS damages but also to primary infection of fetal muscles [41, 42]. Indeed, primary infection of muscle cells was confirmed in ruminant fetuses infected by AKAV [6, 43]. The presence of SBV in muscle tissues is less frequently evaluated, and if so, quite often by IHC or *in situ* hybridization, with frequent negative results [30, 26, 44]. In the current study one muscle sample from a G60 lamb was found positive by RTqPCR, without signs of gross pathology. The lack of SBV positive detection in the serum of any of the lambs excludes a blood origin by the time of the test. In line with this result, Balseiro et al. reported a higher detection level of SBV RNA in muscles than in the brain or spinal cord of malformed calves [45]. These data suggest that primary infection of fetal muscles might act as an auxiliary mechanism to CNS infection in the pathogenesis of the hydranencephaly/arthrogryposis syndrome caused by SBV, as well as speculated for AKAV.

Yet not statistically significant, an intriguing finding was the SBV RNA higher detection rate in meconium from G45 lambs (7/11) than from G60 ones (3/10). Meconium was reported to be a matrix either rarely or frequently found positive by RTqPCR, depending on the considered study [18, 22]. It is noteworthy to underline that at the same time, amniotic fluid was found more often positive in G60 lambs (5/10) than in G45 lambs (2/11) but not statistically significant as well. As meconium is an end-product of the amniotic fluid being swallowed by the fetus during gestation time, it is likely that SBV detection in the meconium could reflect the former transient presence of the virus in the amniotic fluid. Fecal shedding was reported following SBV experimental infection in heifers [46] and ewes [47]. Hence positive SBV RNA detection in meconium might be the *in utero* version of the previously reported positive detection in the feces of adult animals.

## Conclusion

The current study demonstrated a very high placental colonization rate when infection occurred at 45 or 60 days of gestation in an experimental context. As an extension of field data where congenital malformations were highly associated with SBV RNA detection in CNS, the absence of gross pathology evocative of SBV infection was reported along with RTqPCR negative results in CNS. In most of the cases, SBV did not reach CNS or could not produce an infection lasting long enough to be detected at birth. In addition, the lack of reported malformations supports the need of an established SBV infection in the CNS of the fetus to become teratogenic. Quite surprisingly, as the placental barrier along with the increasing immunocompetence were assumed to decrease the likelihood of a positive RNA detection at birth, the closer the infection from the term, the higher was the frequency of organs still positive. SBV could infect the placenta of most of the infected ewes. By contrast, viral genome could only be detected in organs of three new-born lambs, of which only one showed positive detection in CNS. No anti-SBV Abs could be detected before colostrum uptake. These results suggest SBV can readily colonize the placenta, but subsequent infection of the fetus is either rare or short lasting. Consequently CNS infection is even less common and so are malformations, at least following infection of the ewes at 45 or 60 days of gestation. *In situ* hybridization and immunohistochemistry might provide more detailed characterization of the cells involved in placental and fetal colonization. Additional experimental infections of pregnant ewes at earlier times in gestation would allow a further clarification of the limits of the specific stages of gestation leading to congenital defects.
